# Filamin C Deficiency Impairs Sarcomere Stability and Activates Focal Adhesion Kinase through PDGFRA Signaling in Induced Pluripotent Stem Cell-Derived Cardiomyocytes

**DOI:** 10.3390/cells13030278

**Published:** 2024-02-02

**Authors:** Shanshan Gao, Lingaonan He, Chi Keung Lam, Matthew R. G. Taylor, Luisa Mestroni, Raffaella Lombardi, Suet Nee Chen

**Affiliations:** 1 University of Colorado Cardiovascular Institute, University of Colorado-Anschutz Medical and Boulder Campuses, Aurora, CO 80045, USA; shanshan.gao@cuanschutz.edu (S.G.); lingaonan.he@cuanschutz.edu (L.H.); matthew.taylor@cuanschutz.edu (M.R.G.T.); luisa.mestroni@cuanschutz.edu (L.M.); raffaella.lombardi@cuanschutz.edu (R.L.); 2 Department of Biological Sciences, University of Delaware, Newark, NE 19716, USA; lamcg@udel.edu; 3 Department of Advanced Biomedical Sciences, “Federico II” University of Naples, 80138 Naples, Italy

**Keywords:** filamin C, dilated cardiomyopathy, PDGFRA, crenolanib

## Abstract

Truncating mutations in filamin C (*FLNC*) are associated with dilated cardiomyopathy and arrhythmogenic cardiomyopathy. FLNC is an actin-binding protein and is known to interact with transmembrane and structural proteins; hence, the ablation of FLNC in cardiomyocytes is expected to dysregulate cell adhesion, cytoskeletal organization, sarcomere structural integrity, and likely nuclear function. Our previous study showed that the transcriptional profiles of *FLNC* homozygous deletions in human pluripotent stem cell-derived cardiomyocytes (hiPSC-CMs) are highly comparable to the transcriptome profiles of hiPSC-CMs from patients with *FLNC* truncating mutations. Therefore, in this study, we used CRISPR-Cas-engineered hiPSC-derived *FLNC* knockout cardiac myocytes as a model of FLNC cardiomyopathy to determine pathogenic mechanisms and to examine structural changes caused by FLNC deficiency. RNA sequencing data indicated the significant upregulation of focal adhesion signaling and the dysregulation of thin filament genes in *FLNC*-knockout (FLNC^KO^) hiPSC-CMs compared to isogenic hiPSC-CMs. Furthermore, our findings suggest that the complete loss of FLNC in cardiomyocytes led to cytoskeletal defects and the activation of focal adhesion kinase. Pharmacological inhibition of PDGFRA signaling using crenolanib (an FDA-approved drug) reduced focal adhesion kinase activation and partially normalized the focal adhesion signaling pathway. The findings from this study suggest the opportunity in repurposing FDA-approved drug as a therapeutic strategy to treat FLNC cardiomyopathy.

## 1. Introduction

Mutations in filamin C (FLNC) are associated with a broad spectrum of cardiac phenotypes, suggesting different pathogenic mechanisms. Truncation variants are enriched in dilated and arrhythmogenic cardiomyopathies, while missense variants are mainly associated with hypertrophic and restrictive cardiomyopathies [1,2,3,4]. FLNC cardiomyopathy caused by truncating mutations is an inherited disease characterized by interstitial fibrosis, the infiltration of fibrofatty tissue into the left ventricle, and electrical instability. These -pathological features are responsible for the high risk of sudden cardiac death [3,5]. It is known that missense mutations (p.S1624L; p.I2160F) cause restrictive cardiomyopathy via the formation of abnormal protein aggregates [6,7,8]. On the other hand, truncation mutations typically result in a premature stop codon and the loss of expression of the mutant allele (a.k.a. haploinsufficiency). Because of haploinsufficiency, the levels of FLNC are reduced [9], and the phenotype is the consequence of loss-of-function effects. The present study focuses on the identification of pathological mechanisms caused by FLNC haploinsufficiency in the heart.

FLNC is an actin-binding protein and is known to localize to the Z-discs and the intercalated discs (ICDs) [10,11]. FLNC plays an important role in bridging cell–extracellular matrix (ECM) interactions by anchoring the actin cytoskeleton to plasma membrane proteins. Because of its interactions with actin, transmembrane, and Z-disc proteins, the ablation of *FLNC* in cardiomyocytes is expected to dysregulate cell adhesion, cytoskeletal organization, and sarcomere structural integrity. Furthermore, given its ability to interact with multiple molecules, it is plausible that FLNC deletion may affect various intracellular signaling pathways. Moreover, the molecular mechanisms of arrhythmia due to FLNC haploinsufficiency in the heart are largely unknown. Thus, it is critical to understand the roles of FLNC in the pathogenesis of FLNC cardiomyopathy in order to improve the clinical management of these patients.

Homozygous knockout (KO) of *Flnc* in mice is postnatally lethal [12,13]. Our previous study showed that the transcriptional changes, caused by FLNC homozygous deletion in human pluripotent stem cell-derived cardiomyocytes (hiPSC-CMs), are highly comparable to the transcriptome of hiPSC-CMs from patients with *FLNC* truncating mutations [9]. Thus, FLNC homozygous knockout (FLNC^KO^) hiPSC-CMs represent an appropriate human cell model with which to study FLNC biology in the heart and the effects of loss-of-function (LoF) mutations in FLNC cardiomyopathy.

In vitro-cultured cardiomyocytes attach to the coated substratum via focal adhesion (FA), which is structurally similar to the in vivo cardiomyocyte costamere that mediates the cell–extracellular matrix (ECM) interaction and bidirectional mechano-transduction in the heart [14,15]. Focal adhesion kinase (FAK) is a key regulator of the formation and turnover of the FA sites. Furthermore, FAK is known to scaffold signaling molecules involved in cell differentiation and survival [16]. The autophosphorylation of FAK at Tyr397 leads to FA disassembly and ECM degradation via the recruitment of several proteases [16,17,18]. Moreover, FAK is known to phosphorylate the actin-binding domain of α-actinin (ACTN2) and decrease its affinity to actin, leading to sarcomere instability [19]. Although FLNC is known to interact with cell membrane proteins, the effects of FLNC ablation in cardiomyocytes on FA stabilization and its downstream signaling remain largely unknown.

Our RNA sequencing data indicate the significant upregulation of FA signaling and the dysregulation of cardiac thin filament genes in FLNC^KO^ hiPSC-CMs compared to isogenic hiPSC-CMs. Therefore, we aimed to investigate the role of FLNC in sarcomere structural maintenance and the activation of FAK and to identify the downstream molecular mechanisms induced by the activation of FAK upon FLNC deletion. In this study, we used CRISP-CAS9-engineered FLNC^KO^ hiPSC-derived cardiac myocytes as a model of FLNC cardiomyopathy due to haploinsufficiency.

## 2. Materials and Methods

### 2.1. Generation of iPSC Deficient of FLNC Using CRISRP-Cas 9 System

We performed genome-editing on the commercial iPSC line from WiCell® using the CRISPR/Cas9 system from Integrated DNA Technologies, Inc (Coralville, IA, USA). Briefly, RNP complexes were formed in vitro using CAS9 protein, gRNA, and tracer RNA, and delivered into iPSCs via electroporation. Next, the edited iPSCs were isolated from single-cell colonies to ensure homogeneity and two homozygous knockout clones were selected and passed quality control. The single-edited iPSC was sorted into 96 wells and grown into a colony. The edited region/PAM site of FLNC was sequenced and validated via Sanger sequencing. We generated 2 lines of FLNCKO using the same method. Briefly, exomes of FLNC were amplified via a polymerase chain reaction, and Sanger sequencing was performed by using the Big Dye Terminator Cycle Sequencing Ready Reaction Kit on an ABI Genetic Analyzer 3730xl (Applied Biosystems, Waltham, MA, USA) to determine the genetic codes. Genetic variants identified were confirmed in both sense and anti-sense directions in separate sequencing reactions when detected.

### 2.2. Culturing and Cardiac Differentiation of Induced Pluripotent Stem Cells

The iPSCs were seeded onto Matrigel-coated plates after thawing. To coat tissue culture plates, one aliquot (dilution factor) of a Matrigel matrix was resuspended with cold DMEM/F12 and added into plate wells. Plates were swirled gently to evenly distribute the diluted Matrigel matrix and incubated at room temperature for at least 1 hour before use. A coating solution was aspirated from cultureware just before use. The iPSCs were maintained in mTsER plus medium and passaged at around 75–85% confluency. The mTsER medium is changed every other day. The cardiac differentiation of iPSCs was performed based on a previously established protocol [20]. Briefly, differentiation was initiated when iPSCs reached 80% confluency by applying GSK3 inhibitor CHIR99021 in RPMI 1640 medium with a B-27 supplement (without insulin). Wnt signaling inhibitor IWR-1 was added 72 h post differentiation induction. Cells were fed with fresh RPMI 1640 medium with a B-27 supplement (with insulin) starting from day 7, and the medium was changed every 2 days. After 20 days of differentiation from iPSCs, iPSC–cardiomyocytes were sequentially cultured with a glucose-free RPMI 1640 medium and regular RPMI 1640 medium, both with a B-27 supplement (with insulin), to increase the purity of the cardiomyocyte population. For drug treatment, crenolanib with a 100 nM final concentration was used to treat iPSC–cardiomyocytes for 72 h.

### 2.3. Quantitative Real-Time Polymerase Chain Reaction

Total RNA was extracted from iPSC-CMs using a commercially available TRIzol reagent according to manufacturer instructions (Thermo Fisher Scientific, Waltham, MA, USA, Cat#15596026). Total RNA concentration was quantified using a Nanodrop 2000 spectrophotometer (ThermoFisher Scientific). Reverse transcription was performed with a High-Capacity cDNA Reverse Transcription Kit (Applied Biosystems, Waltham, MA, USA). For template amplification, we performed a reaction with the following conditions: 95 °C at 20 s in the denaturation stage, then 40 cycles at 95 °C for 1 s and at 60 °C for 20 s in the holding stage, followed by a melting curve stage. Primers specific to FLNC (sense: 5′-CCTATGCTGTCTCCTATGTG-3′; antisense: 5′-TAGATGTCAAAGTAGGTGGG-3′) were used to determine transcriptional level of FLNC expression. GAPDH (sense: 5′-CTTTTGCGTCGCCAG-3′; antisense: 5′-TTGATGGCAACAATATCCAC-3′) was used as the reference gene for quantification.

### 2.4. Protein Extraction and Immunno Blotting

Proteins were extracted from isogenic control and FLNCKO-/- iPSC-CMs with RIPA buffer (Life Technology, Carlsbad, CA, USA) [9]. Proteins were separated on polyacrylamide gels, and transferred to PVDF membranes (Millipore, Burlington, MA, USA). Membranes were blocked with 5% nonfat milk in TBS-T (Tris-buffered saline, 0.1% Tween 20) at room temperature for 1 h. For protein detection, Phospho-Akt (Ser473) (Cell Signaling, Danvers, MA, USA, Cat#4060, dilution 1:1000), Akt (Cell Signaling, Cat#9272, dilution 1:1000), Phospho-FAK (Tyr397) (Cell Signaling; dilution 1:1000), FAK (Proteintech, Cat#12636-1-AP, dilution 1:1000), and GAPDH (Thermo Fisher Scientific, Cat#AM4300, dilution 1:8000) were used. Membranes were then incubated with horseradish peroxidase-conjugated secondary antibodies and signals were developed using an enhanced chemiluminescence substrate (Thermo Fisher Scientific, Waltham, MA, USA).

### 2.5. Immunofluorescence Staining

Antibodies specific to human pluripotency markers, including TRA160 (MAB4360, 1:100), SSEA4 (BioLegend, San Diego, CA, USA; 330402, 1:100), SOX2 (ThermoFisher: 48–1400, 1:100), and OCT4 (ThermoFisher: C30A3, 1:400), were used in immunofluorescence to confirm the pluripotency of iPSCs. To detect expression and distribution of proteins in iPSC-CMs, cells were replated onto coverslips in a 24-well plate. Then, iPSC-CMs were fixed with 4% paraformaldehyde at room temperature (RT) and permeabilized with 0.1 Triton X-100, followed by blocking with 5% normal donkey serum (Abcam, Cambridge, UK, Cat#7475) and incubation with the following primary antibodies: anti-FAK (Proteintech, Rosemont, IL, USA, Cat#12636-1-AP, dilution 1:800) and anti-Sarcomeric Alpha Actinin antibody (Abcam, Cat#9465, dilution 1:200). The following secondary antibodies were applied: anti-rabbit IgG (H+L), Alexa Fluor™ 488 (Invitrogen, Waltham, MA, USA, Cat# A-11034, dilution 1:1000), anti-rabbit IgG (H+L), and Alexa Fluor™ 594 (Invitrogen, Cat# A-11005, dilution 1:1000). Nuclei were stained with 4′,6-diamidino-2-phenylindole (DAPI). Coverslips were mounted with fluorescence mounting medium (Agilent, Santa Barbara, CA, USA) onto slides and observed under a fluorescence microscope.

### 2.6. Image Analysis

Image analysis was performed using Plugins from ImageJ. The distribution of FAK between cytoplasm and nuclear was analyzed using “Cyt/Nuc” [21]. Briefly, the cytosolic and nuclear immunofluorescence signal were analyzed via an automated quantification algorithm in “Cyt/Nuc” for each cell and nucleus. The profile and spatial locations of the sarcomere was analyzed using fast Fourier Transformation algorithm line profile built-in analysis program according to the instruction by Zeiss Microscopy. Briefly, α-actinin stained images were imported to Zen Blue 3.7 analysis software and line tool was used to profile the distance and signal intensity of the images. The intensity and spatial distribution values were then exported and analyzed using the MAX formula in Excel to obtain the distance measurement. All signal intensity were kept consistent among images for unbiased analysis. All images were analyzed by a second operator to prevent analysis bias.

### 2.7. RNA Sequencing

RNA sequencing was performed on RNA depleted of ribosomal RNA (rRNA) extracted from the iPSC-CMs on an Illumina platform. In brief, total RNA was extracted from the hiPSC-CMs and the concentration of each RNA sample was determined using a NanoDrop Spectrophotometer. RNA samples were then analyzed and sequenced by the University of Colorado Anschutz Medical Campus Genomics Core. Briefly, we performed electrophoretic separation on an Agilent Tapestation 4200 to test RNA integrity and samples with an RNA Integrity Number (RIN) > 8 were used to prepare a sequencing library using the Illumina Tecan/Nugen RNA library preparation kit. The Universal Plus mRNA-Seq library preparation kit with NuQuant was used (Tecan) with an input of 200 ng of total RNA to generate RNA-Seq libraries. Paired-end sequencing reads of 150 bp were generated on NovaSeq 6000 (Illumina) sequencer at a target depth of 80 million paired-end reads per sample. Raw sequencing reads were de-multiplexed using bcl2fastq.The RNA samples were sequenced on the Illumina NovaSeq 6000 with a 2 × 150 bp configuration.

### 2.8. RNA-Seq Data Analysis

For each sample, about 20–30 million pairs of 150 bp pair-end reads were generated. Raw reads were first trimmed for 10 bases at the 5′end to remove reads with biased nucleotide (ACGT) distribution. Trimmed reads were then aligned to the Homo sapiens genome (GRCh38, GENCODE, version 28 and 34, primary assembly) using STAR aligner [22] (version 2.5.0a). Differential gene expression (DEG) analyses counts were performed on the read using DESeq2 [23] (v1.22.2) in R environment. Genes with mean read counts less than 10 from across samples were filtered out from analysis. A gene was considered significantly dysregulated if the adjusted p-values was less than 10% and there was two-fold change based on the shrunken log2 fold change implemented in DESeq2 (FDR < 0.01 and abs(log2FC) > 1). Expression heatmaps were generated using the pheatmap package in R environment. The functional enrichment of the significantly dysregulated genes was carried out using DAVID [24] and gene set enrichment analysis (GSEA) software [25]. The molecular interaction network analysis was performed using Cytoscape [26].

### 2.9. Statistics

Statistical analyses were performed as previously described [27]. Experimental outcomes were expressed as mean ± SD. Statistically significant differences were determined by t-test between two groups and one-way ANOVA among multiple groups, followed by pairwise comparison. Experimental data that did not follow normal distribution were analyzed by the Kruskal–Wallis test. All statistical analyses were performed using GraphPad Prism 9.

## 3. Results

### 3.1. Modeling FLNC-Related Cardiomyopathy Using FLNC Knockout hiPSC-Derived Cardiomyocytes

**Figure 1:**
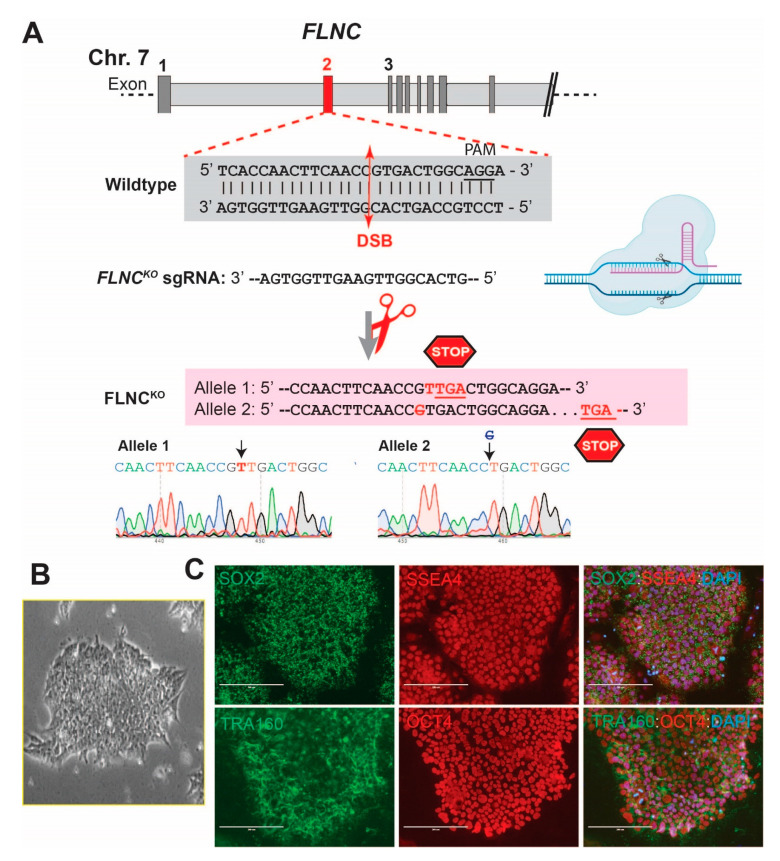
**Generation of FLNC^KO^ hiPSC model of FLNC cardiomyopathy.** (**A**) Top–middle: small guide RNA for CRISPR/Cas9 knockout targeting PAM sequence in exon 2 of *FLNC.* Double-strand breaks induce an insertion/deletion in the targeted PAM region, resulting in an early-stop codon that truncates *FLNC*. Bottom: Sanger sequence chromatograms verify gene editing. (**B**) FLNC^KO^ iPSCs colony showing the typical “spiky” stem cell colony edge morphology (phase contrast image). (**C**) Immunofluorescence staining shows the expression of pluripotency markers (SOX2 (Green), SSEA-4 (Red), TRA160 (Green), and OCT4 (Red)) in FLNC^KO^ iPSCs. ― scale bar representing 200 μm.

Truncating *FLNC* variants associated with dilated and arrhythmogenic cardiomyopathies are loss-of-function (LoF) mutations, causing haploinsufficiency. To recapitulate the human pathogenesis, we generated FLNC-deficient hiPSC lines using the CRISPR/CAS9 system as a model of FLNC-related cardiomyopathy. As shown in Figure 1, we successfully generated FLNC homozygous FLNC^KO^ hiPSC-CMs. The selected clones were characterized via immunofluorescence (IF) to detect stem cell pluripotency markers. Our results showed that FLNC^KO^ hiPSCs exhibited typical stem cell morphology (Figure 1B) and expressed pluripotency markers such as TRA160, SSEA4, SOX2, and OCT4 (Figure 1C).

### 3.2. FLNC Deficiency Causes Structural Remodeling of hiPSC-CMs

**Figure 2:**
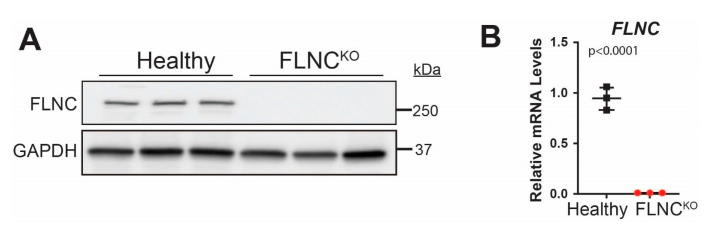
**Verification of FLNC^KO^ hiPSC-CMs.** FLNC^KO^ hiPSC-CMs expressed significantly lower FLNC at both (**A**) protein and (**B**) mRNA levels, as detected by immunoblots and qPCR, respectively.

**Figure 3:**
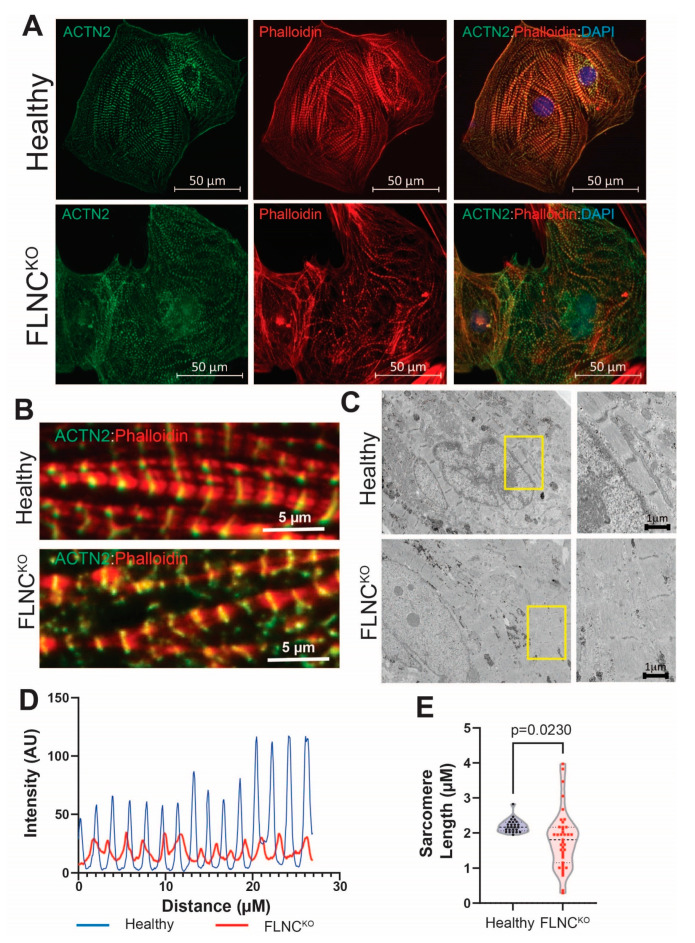
**Sarcomere malformation in FLNC^KO^ hiPSC-CMs.** (**A**) IF images showing α-actinin (Green) and actin (Red) alterations in FLNC^KO^ as compared to isogenic control hiPSC-CMs. (**B**) Zoom-in images showing the irregular organization of thin filaments in FLNC^KO^ as compared to isogenic control iPSC-CMs. (**C**) TEM images of FLNC^KO^, further supporting abnormal organization α-actinin (Green) and actin compared to isogenic control iPSC-CMs. (**D**) Fast Fourier transformation analysis showed the irregular spacing of α-actinin in FLNC^KO^ iPSC-CMs. (**E**) MyofibrilJ analysis showed that the sarcomere lengths of FLNC^KO^ iPSC-CMs were significantly shorter as compared to isogenic control iPSC-CMs.

The expression levels of FLNC were significantly reduced in FLNC^KO^ hiPSC-CMs for both protein and mRNA levels, as detected by immunoblot (IB) analysis and qPCR (Figure 2A,B). We performed immunofluorescence (IF) analysis of actin filaments (F-actin), including both cytoskeleton and sarcomeric thin filaments, using phalloidin and α-actinin (ACTN2, a marker of Z-disc protein and stabilizer of the actin filaments in FA). IF analysis showed actin cytoskeleton disorganization to be associated with an aberrant sarcomere structure in FLNC^KO^ iPSC-CMs (Figure 3A,B). In particular, F-actin and ACTN2 appeared to accumulate in regions where α-actinin was absent. The disruption of the spatial distribution pattern of F-actin and ACTN2 in the FLNC^KO^ hiPSC-CMs suggested reduced crosslinking of the thin filaments of actin to ACTN2, leading to the breakage of actin bundles and disarrangement of protein interactions in the Z-discs. We also confirmed the abnormal structure of the sarcomeres via transmission electron microscopy (TEM), as shown in Figure 3C. The Z-lines in the control hiPSC-CMs appeared as fine dense lines forming defined sarcomere boundaries; conversely, in the FLNC^KO^ hiPSC-CMs, the Z-lines were thinner and interrupted in numerous points. Fluorescent signal analysis using fast Flourier transformation (FFT) showed decreased fluorescent intensity and irregular spacing of ACTN2 in FLNC^KO^ hiPSC-CMs compared to isogenic control hiPSC-CMs (Figure 3D). Also, a significantly wider distribution of sarcomere length (0.2892–3.684 μm vs. 1.948–2.824 μm), with a mean of 1.808 ± 0.8293 vs. 2.202 ± 0.187, was observed in FLNC^KO^ cardiomyocytes compared to controls (Figure 3E). The elongated sarcomere resembled the structural elongation of CMs observed in eccentric hypertrophy. Taken together, the data support the hypothesis that FLNC deficiency causes F-actin mislocalization and the loss of actin-actinin crosslinking, hindering thin filament formation in hiPSC-CMs.

### 3.3. FLNC Deficiency Causes Calcium (Ca^2+^) Handling Abnormalities in hiPSC-Derived Cardiomyocytes

**Figure 4:**
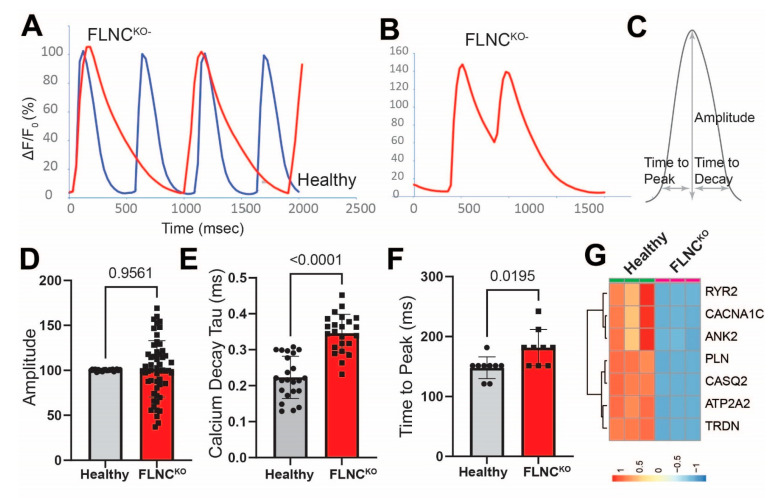
**Calcium (Ca2+) handling abnormalities in FLNC-deficient cardiomyocytes.** (**A**) Calcium handling dysregulations in FLNC^KO^ compared to isogenic control iPSC-CMs. (**B**) Early afterdepolarizations in FLNC^KO^ iPSC-CMs. (**C**) Illustration of calcium transient curve. (**D**) Calcium transient amplitude. (**E**) Calcium decay time. (**F**) Time to peak. (**G**) Calcium handling genes heatmap.

To determine whether structural defects upon FLNC deletion were associated with impaired calcium handling in cardiac myocytes, we performed calcium transient analysis using the calcium indicator dye Cal-520. FLNC^KO^ hiPSC-CMs showed marked calcium handling abnormalities as compared to isogenic control hiPSC-CMs, as illustrated by the representative traces in Figure 4A–C and the quantification data in the bar graphs in Figure 4D–F. Interestingly, 10% of the mutant hiPSC-CMs presented early afterdepolarizations, while none of the isogenic control hiPSC-CMs (EADs) (Figure 4B) did despite being known to trigger ventricular arrhythmias [28,29]. Although there was no difference in calcium transient peak amplitude (Figure 4D) between the two genotypes, FLNC^KO^ hiPSC-CMs displayed significant increases in peak to 50% calcium decay (0.3460 ± 0.0526 vs. 0.2231 ± 0.0586) (Figure 4E) and in time to peak (181.6 ± 30.27 vs. 148.0 ± 18.20 ms) (Figure 4F), indicating the slower reuptake of calcium in the sarcoplasmic reticulum. To delineate the mechanism of altered calcium handling in FLNC^KO^ iPSC-CMs, we determined the expression levels of genes involved in calcium transient cycling from our transcriptome data and found that, in FLNC^KO^ hiPSC-CMs, the mRNA levels of the calcium handling genes, notably the SR/ER calcium ATPase 2a (SERCA), were significantly lower as compared to control isogenic iPSC-CMs (Figure 4G), indicating the dysregulated transcription of calcium handling genes due to FLNC deficiency.

### 3.4. Focal Adhesion Kinase Signaling Pathway Is Upregulated in FLNC-Related Cardiomyopathy

**Figure 5:**
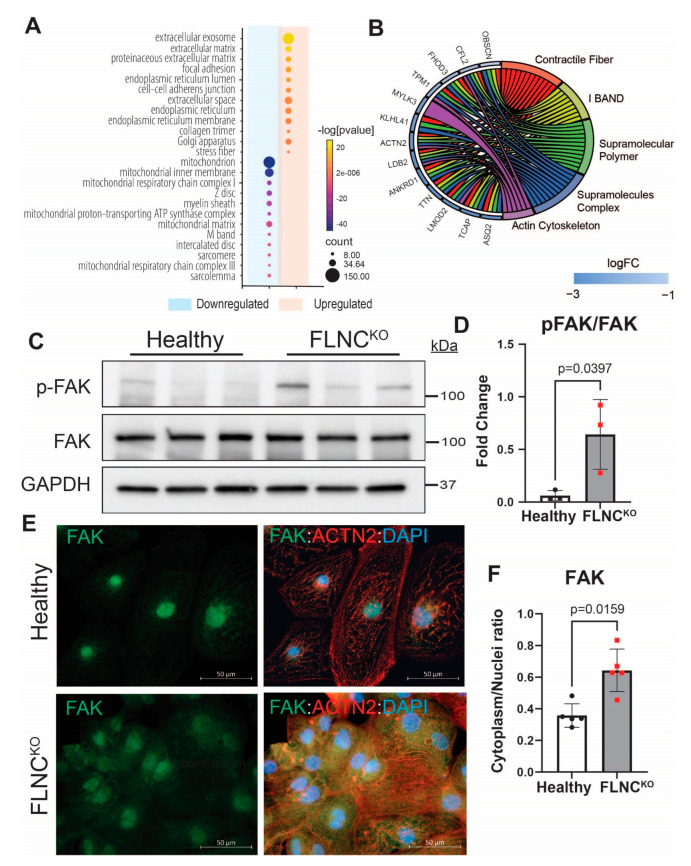
**FAK activation in FLNC^KO^ hiPSC-CMs.** (**A**) GO cellular component analysis in FLNC knockout and isogenic control iPSC-CMs shows the activation of focal adhesion signaling pathway (hsa04510). (**B**) Downregulation of genes involved in sarcomere cytoskeleton formation in FLNC^KO^ iPSC-CMs. (**C**) Increased expression levels of p-FAK (Y397) in FLNC^KO^ compared to wildtype iPSC-CMs as assessed by IB. (**D**) Quantitative representation of the blots in B. (**E**) IF stanning of FAK in FLNC^KO^ compared to isogenic control iPSC-CMs. (**F**) Quantitative representation of the fluorescence signal of FAK in cytoplasm and nucleus.

RNA sequencing bioinformatics analysis demonstrated the dysregulation of genes involved in molecular signaling and cellular processes at the plasma membrane in FLNC^KO^ hiPSC-CMs (Figure 5A). Notably, the expression profile of RNA-seq data from FLNC^KO^ hiPSC-CMs showed the activation of the focal adhesion signaling pathway (hsa04510) and the downregulation of genes involved in the formation of the sarcomeres and intercalated discs. Circle plot analysis of the downregulated genes revealed that these genes were involved in non-covalent polymerization and macromolecule interactions, the biochemical processes governing the formation and crosslinking of contractile fibers and actin cytoskeleton (Figure 5B). To validate the transcriptome findings of FA pathway activation, we performed IB and detected the significantly increased expression levels of the active p-FAK (Y397) in FLNC^KO^ compared to healthy hiPSC-CMs (Figure 5C,D). Also, we performed IF to detect the localization of FAK and found a significant increase in cytosolic FAK, as indicated by higher FAK cytoplasm/nuclei ratio (0.3580 ± 0.0741 vs. 0.6432 ± 0.1346) in FLNC^KO^ compared to isogenic control hiPSC-CMs (Figure 5E,F). In addition, FLNC^KO^ hiPSC-CMs presented significantly decreased levels of intercalated disc (ID) proteins (as previously published by our group) and sarcomeric genes compared to isogenic hiPSC-CMs. Taken together, the data support the notion that FLNC haploinsufficiency activates focal adhesion signaling, which leads to structural impairment of the cytoskeleton, intercalated discs, and sarcomeres.

### 3.5. Inhibition of PDGFRA Signaling Attenuates FA Signaling Pathway Activation in FLNC^KO^ Cardiomyocytes

**Figure 6:**
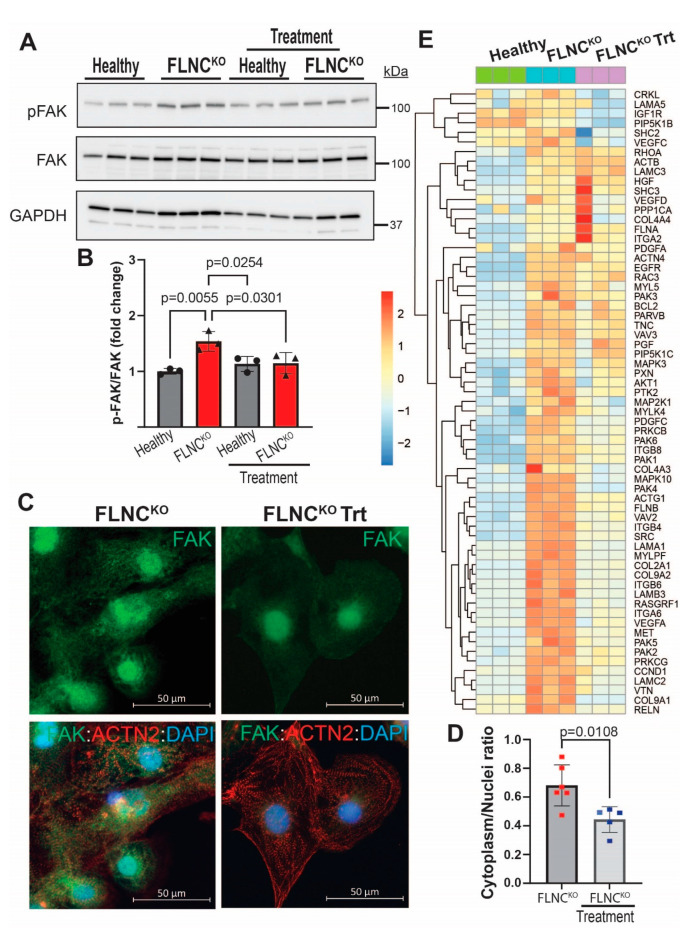
**PDGFRA inhibition by crenolanib-rescued FAK signaling.** (**A**) Crenolanib treatment attenuated FAK phosphorylation in FLNC^KO^ compared to isogenic controls iPSC-CMs. (**B**) Quantitative representation of the IB blots in (**A**). (**C**) Reduced cytoplasm FAK localization by IF. (**D**) Quantitative representation of the fluorescence signal of FAK in cytoplasm and nucleus in FLNC^KO^ hiPSC-CMs before and after treatment. (**E**) GSEA analysis showing partial normalization of DEGs in the FA pathway (hsa04510).

We have previously shown that activation of the PDGFRA signaling pathway is a likely pathogenic signaling regulator in the pathogenesis of FLNC-related cardiomyopathy and that treatment with crenolanib, an FDA-approved PDGFRA inhibitor, attenuates the arrhythmic phenotype of FLNC^KO^ hiPSC-CMs [9]. To test the hypothesis that PDGFRA is an upstream regulator of FAK signaling, we pharmacologically inhibited PDGFRA with crenolanib and determined the effects of the treatment on FAK phosphorylation status. PDGFRA inhibition by crenolanib reduced the phosphorylation of FAK in FLNC^KO^ compared to isogenic control hiPSC-CMs (Figure 6A,B). The reduced p-FAK expression levels were further supported by reduced localization of FAK in the cytoplasm, as shown by IF (Figure 6C). Gene set enrichment analysis (GSEA) revealed the partial normalization of the FA pathway (hsa04510) in the FLNC^KO^ hiPSC-CMs upon pharmacological inhibition of PDGFRA signaling, supporting the hypothesis that PDGFRA activation dysregulates FAK in FLNC^KO^ hiPSC-CMs (Figure 6D).

**Figure 7:**
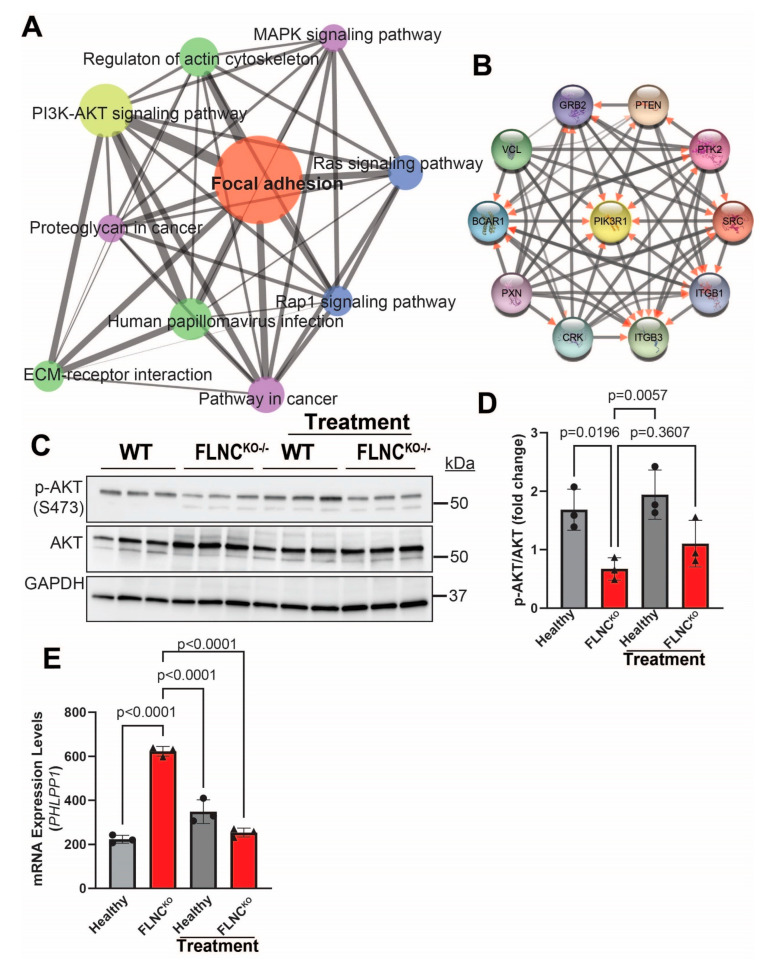
**Suppression of AKT signaling in FLNC^KO^ iPSC-CMs.** (**A**) Enrichment of PI3K-AKT signaling pathway, MAPK signaling (*p* = 3.09 × 10^−80^) pathway, and actin cytoskeleton DEGs in FLNC^KO^ compared to isogenic control iPSC-CMs. (**B**) The molecular interaction network analysis revealed PIK3 kinase regulatory subunit 1 (PI3KR1) to be the core molecule. (**C**) Immunoblots showed decreased p-AKT expression levels in FLNC^KO^ iPSC-CMs compared to isogenic control iPSC-CMs; total AKT expression levels were also significantly increased compared to isogenic control iPSC-CMs. (**D**) Quantitative representation of immunoblots in C. (**E**) *PHLPP1* mRNA was increased in FLNC^KO^ compared to healthy hiPSC-CMs, and treatment with crenolanib significantly downregulates *PHLPP1* mRNA levels. All experiments were independently repeated three times.

To determine the downstream effectors of the FAK signaling pathway, we compared the transcriptomes of FLNC^KO^ hiPSC-CMs with isogenic control hiPSC-CMs to identify potential pathways that are associated with focal adhesion dysregulation due to FLNC haploinsufficiency. GSEA analysis using the KEGG pathways database (Figure 7A) showed that the dysregulated genes involved in FA signaling pathway in FLNC^KO^ hiPSC-CMs were enriched for PI3K-AKT (*p* = 3.09 × 10^−80^) and MAPK (*p* = 3.09 × 10^−80^) signaling pathways, as well as for the regulation of actin cytoskeleton (*p* = 1.33 × 10^−60^) (Figure 7A). Network analysis using Cytoscape^®^ revealed PIK3 kinase regulatory subunit 1 (PI3KR1) to be the core molecule of the molecular network, further supporting the notion that PI3K-AKT signaling pathway is a plausible downstream effector of FAK. However, the immunoblots in Figure 7C,D show that p-AKT, the active form of AKT, levels were significantly reduced, while the total levels of AKT were significantly increased in FLNC^KO^ hiPSC-CMs compared to isogenic control hiPSC-CMs. This finding suggests that AKT activity is reduced in our model. To investigate the mechanisms leading to lower p-AKT expression levels, we determined the levels of PH domain leucine-rich repeat protein phosphatase (PHLPP1), a selective phosphatase of AKT known to selectively dephosphorylate AKT at Ser473. Interestingly, the transcripts of PHLPP1 exhibited a 3-fold increase in FLNC^KO^ hiPSC-CMs compared to isogenic control hiPSC-CMs. Taken together, the findings suggest that FAK is a downstream effector of PDGFRA and that AKT is suppressed due to upregulation of PHLPP1 in FLNC-deficient hiPSC-CMs.

## 4. Discussion

**Figure 8:**
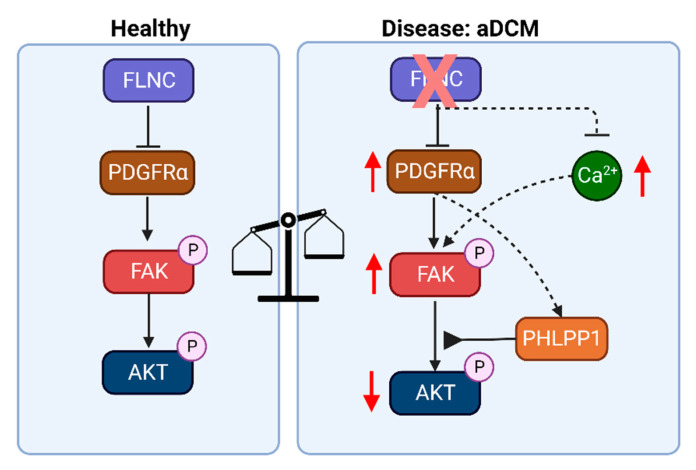
**FAK signaling in FLNC cardiomyopathy.** Our data indicate that FLNC is an important regulator of sarcomere structural stability in cardiac myocytes. The loss of FLNC causes structural defect, the activation of pathogenic PDGFRA/FAK signaling pathways, and the downregulation of AKT signaling, which is important for cardiac growth. In this study, we found that AKT activation was reduced with the expression levels of PHLPP1, an AKT-specific phosphatase. The PHLPPA transcript levels were increased in FLNC^KO^ iPSC-CMs compared to isogenic healthy iPSC-CMs. Therefore, we hypothesized that the deletion of FLNC in iPSC-CMs upregulated the expression levels of PHLPP1, a finding that needs further investigation. --- broken line arrows indicate possible/alternative hypothesis.

FLNC is an actin-binding and crosslinking protein with an important role in maintaining the structural integrity of Z-discs, intercalated discs, and cell membranes of cardiomyocytes [30,31,32]. In this study, we showed that the complete loss of FLNC dysregulates the assembly of thin filament bundles and the focal adhesion signaling pathway (Figure 8). The thin filament disorganization observed by IF in our FLNC^KO^ hiPSC-CMs indicates aberrant sarcomere organization, which is also evidenced by the transcriptomic profile of genes involved in Z-disc biological functions (Figure 5A,B). The activation of the focal adhesion pathway is likely caused by the structural sarcomere and cytoskeleton defects and calcium transient dysregulation, as observed in the FLNC^KO^ hiPSC-derived cardiomyocytes.

The sarcomere structural abnormalities observed in our hiPSC-CMs upon FLNC ablation are consistent with the findings of other research groups [8,13]. Interestingly, the FLNC^KO^ hiPSC-CMs also show decreased mRNA expression of cardiac thin filament genes such as ACTC1, ACTN2, TNNC1, TNNI3, TNNT2, and LMOD2. The transcriptional dysregulation of these genes indicates that FLNC plays an important role in regulating thin filament formation in cardiomyocytes, from transcription to the crosslinking of polymeric actin and macromolecule assembly in the mature thin filaments. Ultimately, the loss of FLNC-mediated actin-crosslinking and deficient thin filament gene expression causes the severe impairment of both contractility and relaxation in FLNC^KO^ hiPSC-CMs. These effects explain the embryonic lethality of homozygous null mice [13] and the fact that thus far no homozygous null alleles have been described in humans.

Calcium homeostasis plays an essential role in maintaining excitation–contraction (E-C) coupling in cardiomyocytes. Th disruption of calcium homeostasis in cardiomyocytes is a major contributor to arrhythmias and heart failure. In cardiomyocytes, transient increases in cytosolic calcium induce contraction during E-C coupling, followed by calcium’s return to the sarcoplasmic reticulum using the Sarcoendoplasmic Reticulum Calcium ATPase (SERCA), which induces muscle relaxation. In cardiomyocytes, SERCA is the dominant calcium transport protein that contributes to the decay of calcium transients. In this study, we found that the expression levels of SERCA were lower in FLNC^KO^ compared to isogenic control cardiomyocytes, but further studies are necessary to determine the mechanisms of longer calcium decay times after FLNC deletion. FLNC deficiency may affect calcium cycling through several mechanisms, cytoskeletal disorganization may affect the structure of the sarcoplasmic reticulum, while the disarrangement of the sarcomere may affect the calcium sensitivity of the thin filaments. Both of these abnormalities may induce negative feedback upon calcium handling gene expression. Similarly to our results, previously published studies showed that the loss of plakophilin-2 (PKP2), a causal gene of arrhythmogenic cardiomyopathy, suppresses the transcription of genes that control calcium cycling [33]. PKP2 is smaller than FLNC and it is known to migrate to the nucleus, and so it is plausible that it could have a direct effect on gene transcription. On the other hand, FLNC interacts with multiple partners and its effect on transcription may be indirect, working through interaction with nuclear membrane proteins, which may ultimately affect chromatin status. Our study raises the need for a better understanding of the role of FLNC in the regulation of calcium transport proteins in cardiomyocytes and consequently the pathogenesis of arrhythmias and reduced contractility in FLNC-related cardiomyopathy. Furthermore, our findings suggest that FLNC may regulate gene expression by affecting chromatin remodeling.

Focal adhesion is a dynamic structure with a high turnover rate that is implicated in cellular differentiation and migration, as well as sarcomere formation and organization, during cardiac development [34,35]. FAK is a nonreceptor tyrosine kinase that functions as an activator of the FA [36]. Increased cytosolic Ca^2+^ has been shown to trigger local disassembly of FAs in human U87 astrocytoma cells through the phosphorylation of FAK at Tyr^397^ [37]. Furthermore, FAK activation has been shown to cause cardiomyopathies [38,39,40].

Recent studies suggest that PI3K/Akt signaling plays a role in the homeostatic regulation of Ca^2+^ in heart failure [41]. In our study, we showed that the calcium decay time was increased in the FLNC^KO^ hiPSC-CMs compared to the isogenic control hiPSC-CMs and that FAK was activated; however, we could determined whether the abnormalities in Ca^2+^ transients were the consequence of the effects on calcium handling genes by FLNC deletion or the impact of FAK activation on calcium cycling genes. Further studies are necessary to delineate mechanisms of Ca^2+^ signaling dysregulation in FLNC cardiomyopathy.

AKT signaling is required for physiological cardiac growth and it is an important antagonist of pathological remodeling in cardiac disease [42]. In cardiac myocytes, AKT1 positively induces Ca^2+^ entry through L-type calcium channels, increases SERCA2 protein levels, and phosphorylates phospholamban [43,44,45]. Our findings suggest the dysregulation of AKT and PHLPP1 contributes to the pathogenesis of FLNC cardiomyopathy. It is plausible that AKT inactivation in our FLNC^KO^ hiPSC-CMs is due to the upregulation of PHLPP1, a selective AKT phosphatase. Protein phosphatase 2A (PP2A) and PHLPP1 have been shown to dephosphorylate Akt at Ser473, resulting in the inactivation of the AKT signaling [46,47,48]. In our study, PHLPP1 transcript levels were significantly increased (Figure 7E) while PP2A levels were decreased (1827 ± 111.9 vs. 3575 ± 271.0; *p* < 0.0001) in FLNC-deficient hiPSC-CMs, suggesting that PHLPP1 is selectively upregulated in the context of loss of FLNC in blunting AKT signaling in cardiomyocytes. Moreover, our findings indicate that PHLPP1 is a target of crenolanib. This is because PHLPP1 transcripts were significantly attenuated, while pAKT expression levels were upregulated (in trend), and PP2A transcripts remained unchanged (1827 ± 111.9 vs. 1890 ± 292.9; *p* = 0.9671) upon treatment with crenolanib. However, further studies are required to reveal the role of AKT and PHLPP1 in the pathogenesis of FLNC cardiomyopathy as well as to repurpose crenolanib to treat FLNC cardiomyopathy.

Our previous study showed that the inhibition of PDGFRA also partially rescued beta-catenin localization. However, from this study, we could not make a conclusion as to whether beta-catenin is upstream or downstream of the FAK signaling pathway. From the literature, there is no doubt that FAK crosstalks with the Wnt/beta-catenin signaling pathway and that the activation of the FAK signaling pathway promotes the activation of beta-catenin by phosphorylating GSK3-beta (an inhibitor of beta-catenin). This result is consistent with our finding where beta-catenin localized to nuclei of FLNCKO hiPSC-CMs, indicating beta-catenin activation. Therefore, we could possibly conclude that FLNC deletion concordantly activated FAK and beta-catenin signaling pathways. However, further studies are needed to determine if FAK is an upstream regulator of beta-catenin in hiPSC-CMs, such as using FAK inhibitor to investigate the nuclear localization of beta-catenin in FLNC-deficient hiPSC-CMs.

## 5. Conclusions

In conclusion, our findings suggest that the complete loss of FLNC in cardiomyocytes leads to cytoskeletal and sarcomeric defects, calcium handling dysregulation, and the activation of focal adhesion kinase. Moreover, our findings show that inactivation of AKT signaling contributes to the pathogenesis of FLNC cardiomyopathy. The inhibition of PDGFRA signaling using crenolanib reduces FAK activation and partially normalizes differentially expressed genes in FA signaling pathways, suggesting that the repurposing of crenolanib may be an effective treatment in FLNC cardiomyopathy.

## 6. Novelty and Significance

What is known?

FLNC is a muscle-specific isoform of the filamin family and is an actin-crosslinked protein that localizes primarily at sarcomere Z-discs.

Exposure to FLNC homozygous variants that cause the loss of FLNC protein expression results in early lethality.

FLNC truncating variants are associated with dilated cardiomyopathy.

What new information does this article contribute?

FLNC homozygous knockout profoundly disrupted Z-disc and thin filament formation in human iPSC-derived cardiomyocytes (hiPSC-CMs), resulting in activation of FAK.

Treatment with crenolanib blunted FAK activation in FLNC^KO^ iPSC-CMs.

## Data Availability

All data needed to evaluate the conclusions in the paper are present in the paper. The human iPSC models in this study can be provided by Dr. Luisa Mestroni and Dr. Suet Nee Chen pending scientific review and a completed material transfer agreement. Requests for the iPSC models should be submitted to Dr. Suet Nee Chen: suet.chen@cuanschutz.edu.
